# Results from the 2013 Senior’s Health Services Survey: Rural and Urban Differences

**DOI:** 10.4172/2471-9846.1000213

**Published:** 2018-03-08

**Authors:** Janis E Campbell, Amanda E Janitz, Keith Kleszynski, Claire Dowers-Nichols, Amber S Anderson, Andrew N Dentino, Laurence Z Rubenstein, Thomas A Teasdale

**Affiliations:** 1 College of Public Health, University of Oklahoma Health Sciences Center, 801 NE 13th St., Oklahoma City, OK, 73104, USA; 2 Donald W Reynolds Department of Geriatric Medicine, University of Oklahoma Health Sciences Center, 1122 NE 13th St., Oklahoma City, OK, 73117, USA

**Keywords:** Survey, Activities and services, Recruitment, Urban, Rural

## Abstract

**Purpose:**

The purpose of this study was to compare and contrast health education needs of rural Oklahomans aged 65 and older compared to urban and sub-urban populations.

**Methods:**

Surveys were distributed to a list of registered voters age 65 and older in Oklahoma with a total of 1,248 surveys returned. Survey items asked about interests in services, classes and activities, plus current barriers to accessing and/or engaging in such programs.

**Findings:**

Survey respondents living in large rural towns (23.7%) and the urban core (21.5%) were significantly more likely than those in small rural towns (14.0%) or sub-urban areas (15.5%) to have attended a free health information event in the past year (*P*=0.0393). Older Oklahomans in small towns and isolated rural areas reported more frequently than those in the urban core that they would participate in congregate meals at a center (small town/isolated rural: 14.4%, urban core: 7.2%) (*P*=0.05). Lack of adequate facilities was more frequently reported by those residing in small town and isolated rural areas compared to urban core areas (16.4% *vs.* 7.8%, *P*=0.01). Finally, older Oklahomans in the large rural towns (0.6%) and small town and isolated rural locations (2.13%) less frequently reported use of senior information lines (Senior Infoline) than those in the urban core (6.0%) and in sub-urban areas (7.1%) (*P*=0.0009).

**Conclusions:**

Results of this survey provide useful data on senior interests and current barriers to community programs/activities have some unique trends among both urban and rural populations.

## Introduction

The 2015 American Community Survey estimated that there were 576,031 (14.7%) individuals aged 65 and older living in Oklahoma. The number of seniors in Oklahoma is expected to increase almost fifty percent to more than 757,000 older Oklahomans by 2030 [1,2]. In addition, Oklahoma’s health indicators continue to be among the poorest in the U.S. According to the United Health Foundation, Oklahoma ranked 48th in “overall senior health” in 2017 [3]. Thus, the need for Oklahoma’s older population to participate in health education and promotion services, activities and programs is critical. Moreover, we know that rural populations often, although not always, have increased health risks including decreased access to care, decreased survival and chronic health conditions [4–7].

A wide array of health education and health activities has been shown to improve senior health [8–13]. Moreover there is a recent focus on improving health literacy to improve health outcomes [8,14–19]. Having the capacity to obtain, process and understand basic health information and services needed to make appropriate health decisions is critical for older adults to be engaged in preserving their health and decreasing disparities in health outcomes [20–27]. In a reciprocal manner, education and service providers must be selective in what activities to offer due to limited time, funds and interest from consumers. Activities that address risk factors and help individuals both avoid and cope with disease are highly valued by older adults.

A few studies have focused on rural populations and older consumer needs [13,28–39]. One early study by Scala [28] reported that “older people living in rural areas face unique challenges, not only in accessing benefits and services, but also in gathering information about programs that can help them.” Rural and inner city areas have decreased access to services in New York State [30]. In a retrospective chart reviews (2004) rural patients were less likely to meet hemoglobin A1c and low-density lipoprotein cholesterol goals, less likely to receive screening and preventative services such as lipid profiles, eye examinations, microalbumin screening, aspirin therapy and vaccinations as compared to urban patients [40]. Rural patients experiencing a cardiac event in British Columbia were more likely to report transportation problems and a lack of local resources and community support for their post-treatment care [41]. Finally, rural people in Appalachian North Carolina were less likely to get regular check-ups and to receive care for chronic conditions [42,43] and those in rural Vermont had decreased physician visits [44].

The growing senior population and their increasing need for health care, the evidence for health education and promotion effectiveness and the lack of information available to guide development of these services, particularly in rural areas, prompted the University of Oklahoma and the Donald W. Reynolds Foundation to initiate the Oklahoma Healthy Agency Initiative (OHAI). The aim of the Oklahoma Healthy Aging Initiative is to, “enhance the health and quality of life for Oklahoma’s seniors by increasing access to geriatric healthcare, providing excellence in health education and optimizing health and aging policy” [45]. This statewide program uses a three-prong approach to improve the wellness of seniors: 1) Increase access to and quality of interdisciplinary geriatric healthcare 2) Provide excellence in health education to healthcare professionals, students of the healthcare and social service disciplines, older adults and their families and the community at large and lastly Optimize health and aging policy. OHAI’s five Centers of Healthy Aging provide both clinical care and health education throughout Oklahoma. One of the first tasks undertaken by OHAI was the 2013 Consumer Needs Assessment Survey (CNAS), which was implemented to determine the health education and caregiving needs of Oklahoma citizens aged 65 and older. The purpose of this study was to evaluate interest in services, classes and activities among rural seniors compared to their urban and sub-urban counterparts that OHAI could potentially offer to seniors living in Oklahoma.

## Methods

### Sample

Data were collected by a mailed survey to a stratified random sample of all 475,518 registered voters age 65 and older in Oklahoma. Details of the survey design and weighting scheme are published elsewhere and will be reported here only briefly [46]. We obtained the Oklahoma voter’s registration file, current as of January, 2013. This file, purchased from the Oklahoma State Election Board, contains information on all registered voters in Oklahoma and includes voter name, address, date of birth and mailing address by county of registration. Using the estimated population counts from the US Census from 2011 and accounting for deceased individuals on the voter registration rolls, we estimated that approximately 85% of all Oklahomans aged 65 and older were represented in the data. A study of voting and registration in the election of November 2012 showed that 87.4% of Oklahomans age 65–74 and 66.5% of Oklahomans age 75 and older were registered to vote [47].

### Instrument-survey information

**Figure 1:**
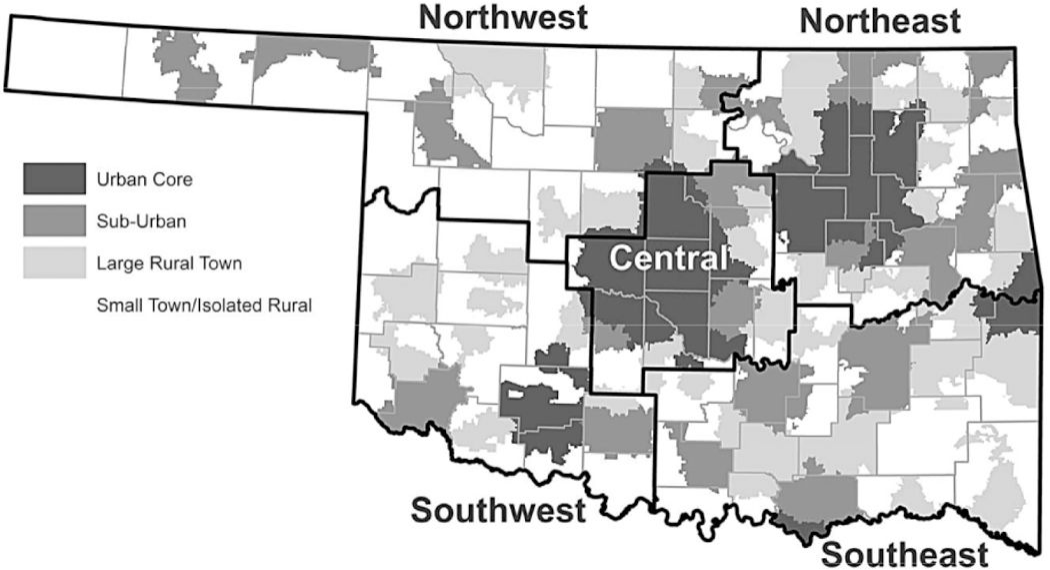
Oklahoma’s five OHAI Regions.

The survey was mailed to a stratified random sample of older Oklahomans, with the strata being Oklahoma’s five OHAI Regions (Figure 1). This assured an adequate sample size for each geographic area within the state (stratum), including both rural and urban areas. The survey was anonymous; thus responses were not traceable to any individual or to the originally mailing list. However, gender, age and ZIP code were requested which allowed us to further stratify results by age and region. Each survey packet included an eight-page paper survey and a self-addressed postage paid return envelope. Surveys were mailed on April 23, 2013 (n=6,705) (Figure 1).

Overall methodology for this survey was described in Campbell et al. [46]. The survey’s six sections included questions concerning current daily activities and transportation issues, current attendance at and interest in health information events, interest in services, classes, or activities for health improvement, current sources of information and assistance concerning health promotion programs, caregiving and basic demographic information. Demographic variables were collected without sacrificing anonymity and no personal health information was recorded. The design allowed for analysis by demographic variables, delineation of interests in a variety of health promotion offerings (including check lists and open-ended responses) and break-out by services, classes and activities. Poverty level was assigned from U.S. Census data and applied to respondents whose ZIP codes were reported (unknown n=99). Each respondent was assigned an aggregate poverty level category based on the percentage of the population in the respondent’s ZIP code with income below the federal poverty level (FPL) (<5%, 5–9%, 10–14%, >15%).

### Classification of metropolitan status

Rural-urban areas were determined from ZIP codes using the four-tier consolidation of the Rural Urban Commuting Area Codes (RUCA) system [48,49]. For general descriptive analyses where subcounty data are available, the four-tiered approach based on secondary codes seemed to allow the most analysis as the lowest geographic level (Figure 1). The four-tiered system includes: 1) urban core (contiguous built-up areas of 50,000 persons or more corresponding to US Census Bureau’s Urbanized Areas); 2) sub-urban areas (often in metropolitan counties, with high commuting flows to urban cores); 3) large rural town (includes towns with populations between 10,000 and 49,999 and surrounding rural areas with 10% or more primary commuting flows to these towns, as well as secondary commuting flows of 10% or more to urban cores); and 4) small town and isolated rural areas (includes populations below 10,000 and their surrounding commuter areas and other isolated rural areas with more than one hour driving distance to a nearest city) (Figure 1).

### Data analysis

Because we used a stratified sampling method to generalize our results to the entire population of Oklahoma aged 65 and older, our estimates were weighted by age and region. We used weights that accounted for the probability of being included in the sample by taking the inverse of the proportion of non-response due to returned mail (1/(Returned Mail/Voter Sample Population)). By applying weights to each response we were able to complete statewide estimates. All percentages and standard errors (SE) were weighted. To account for survey weighting, Rao-Scott Chi-Square Tests were performed to determine differences.

We calculated frequencies, weighted percentages and weighted SEs for the survey questions related to services, classes and activities that were of interest to older Oklahomans (65 and older) if available free of charge or at a significantly reduced rate, stratified by RUCA status. The continuous variable of travel distance for necessities such as groceries or prescriptions was analyzed using a weighted t-test. All analyses were conducted using SAS^®^ 9.4 (Care, NC). We assumed an alpha of 0.05 unless otherwise specified. The study was approved by the Institutional Review Board at the University of Oklahoma Health Sciences Center.

## Results

A total of 1,248 surveys were returned and analyzed, representing a 19.8% response rate [46]. Survey response rates varied by OHAI region the lowest response rate being for the Southwest region [46]. Additionally, survey responses varied by RUCA areas (Table 1) with the most important factor being the very low response rates among the sub-urban areas (5.9%) and the somewhat high percentage of unknown ZIP codes, thus unknown RUCA codes (7.9%) (Table 1).

### Overall

**Figure 2:**
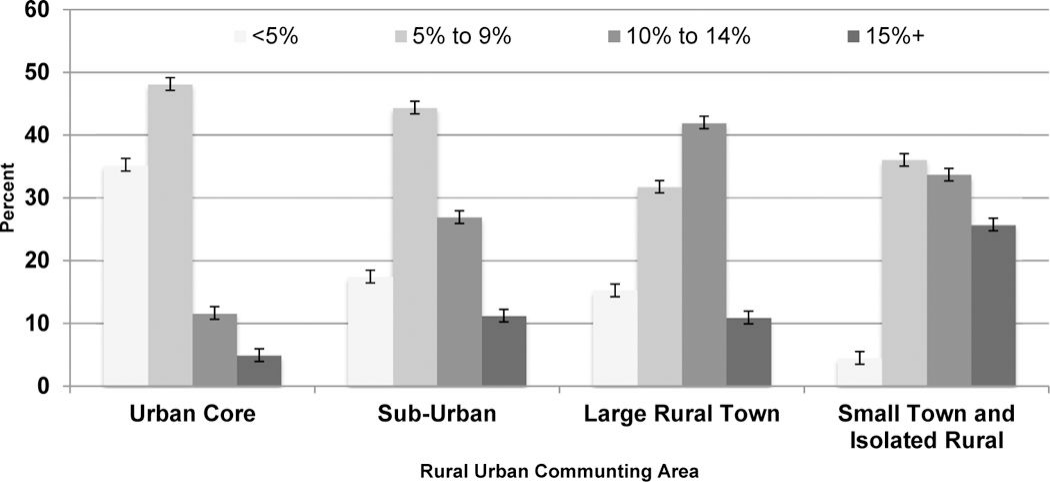
Percent of the area population below the FPL by RUCA status.

We observed no differences by gender (*P*=0.21) or age groups (*P*=0.71) by RUCA (data not shown). Oklahomans living in large rural towns and the urban core (8.3% and 6.3%, respectively) were more likely than those living in the small town and isolated rural areas (4.1%) to use walking or bicycles/tricycles as transportation (*P*=0.009) (Table 2). Older adults in the urban core drive an average of 5.5 miles for necessities such as groceries or prescriptions, while those in all other geographic areas drive longer average distances (large rural towns: 11.0 miles (*P* <0.0001); sub-urban areas: 13.0 mile (*P* <0.0001); small town/isolated rural areas: 14.2 miles (*P*<0.0001). Seventy-seven percent (77.0%) of older Oklahomans who live the urban core were reported residing within five miles of groceries or prescriptions compared to only 28.0% of those in the sub-urban, 54.3% of those in large rural towns and 37.5% of those in small town and isolated rural areas (*P*<0.0001). We observed a significant difference (*P*<0.0001) in the percent of the area population below the FPL by RUCA status (Figure 2). High poverty levels (with 15% or more of the population being below the FPL) are highest in the small towns and isolated rural (25.8%, SE 2.44), in the middle in both the sub-urban and large rural towns (11.2%, SE 3.06 and 11.0%, SE 2.25 respectively) with the urban core being the lowest (4.9%, SE 1.2).

We observed differences in attendance at free health information events in the past year (*P*=0.04), with respondents living in large rural towns (23.7%) and the urban core (21.5%) reporting attendance more frequently than those in small rural towns (14.0%) or sub-urban areas (15.5%) (Table 2). One important difference among the RUCA was that older Oklahomans living in sub-urban areas rarely stated I don’t leave my home as where they spent most of their time away from home (0.7%) compared to all other RUCA (urban core 7.0%, large rural towns 4.3% and small town and isolated rural 6.3%) (*P*=.02) (Table 2).

### Services/activities/classes

When asked about their interest in using services, classes or activities if they were available free of charge or for a significantly reduced rate, we observed few differences (Table 3). Older Oklahomans in small towns and isolated rural areas reported more frequently than those in the urban core to say they would participate in congregate meals at a center (small town/isolated rural: 14.4%, urban core: 7.2%) (*P*=0.05).

### Perceived barriers to services

The most common answer for perceived barriers to accessing programs was *just don’t want to go*, though this differed only marginally by RUCA status (*P*=0.05). Among those in large rural towns (33.9%), small town and isolated rural (31.6%) and the urban core (26.0%) had the highest responses with sub-urban (19.9%) being the lower. Lack of adequate facilities was more frequently reported by those residing in small town and isolated rural areas compared to urban core areas (16.4% *vs.* 7.8%, *P*=0.01). Transportation was reported as a problem more frequently in the urban core (10.9%) than sub-urban (5.2%), small town and isolated rural (4.1%) and large rural towns (4.0%) (*P*=0.004) (Table 4).

### Sources of information about community programs

Older Oklahomans from the urban core area reported a higher frequency of using aging agencies, senior centers, or retirement communities to find out information about help for older adults (urban area: 21.5%, sub-urban area: 6.3%, large rural town: 14.6%, small town/isolated rural: 14.4%, *P*=.0008) (Table 5). Older adults, living in large rural towns (33.8%) and the urban cores (34.4%) more frequently reported that they find information from churches than those in small town and isolated rural locations (24.94%) or sub-urban areas (25.8%) (*P*=0.04). Those residing in small town and isolated rural areas (18.8%) and sub-urban areas (20.6%) less frequently reported that they accessed a national organization such as AARP for help than those in large rural towns (26.4%) and the urban core (28.9%) (*P*=0.03). Finally, older Oklahomans in the large rural towns (0.6%) and small town and isolated rural locations (2.13%) less frequently reported use of senior information lines (Senior Infoline) than those in the urban core (6.0%) and in sub-urban areas (7.1%) (*P*=0.0009).

In addition to resources for help, we also observed differences in how residents found information in their community by RUCA status (Table 5). Older adults in the urban core (58.6%) reported less frequently using family, neighbors, or friends as a source of community information than those living in the sub-urban area (68.2%), large rural town (72.1%) or small town and isolated rural areas (74.9%) (*P*=0.0002). Those living in the urban core (33.0%) more frequently reported using the internet than those in sub-urban (26.4%), large rural town (27.0%, or small town/isolated rural areas (19.4%) (*P*=0.005). Those living in the urban core (45.7%) more frequently reported using newsletters, flyers or bulletins than those in sub-urban (40.0%), large rural town (42.9%), or small town/isolated rural areas (33.9%) (*P*=0.04). Lastly, older adults living in the urban core (72.0%) more frequently reported using television to find out what was happening in their community than those living in sub-urban (56.1%), large rural town (62.2%), or small towns and isolated rural areas (51.5%) (*P*<0.0001).

## Discussion

Results of this survey provide useful data on older adults’ general demographic trends, desires for services, classes and activities as well as perceived barriers to community programs/activities in urban, sub-urban, large town and small town/isolated rural populations (Table 6). One in five older adults attended an event offering free health services in 2013. Older adults in Oklahoma clearly (in virtually all subgroups) reported being interested in services that include legal assistance, health screenings, assistance with tax preparation and prescription assistance. For the most part, there were no differences in these populations by geographic area.

However, there are a few important difference between those older adults located in specific rural or urban areas. The major demographic difference was that small town and isolated rural populations tended to have higher poverty, which is often further complicated by transportation [41]. In our study, we observed that transportation was actually more likely to be perceived as a problem in the urban core areas (10.9%) as compared to more rural areas (5.2% or less). Similar to other studies we observed that outside of the urban core, a lack of adequate facilities was reported as a problem to accessing programs and resources [28,30,40–43].

Perhaps one of the more interesting findings from this study was in understanding the methods that seniors get their information, with clear differences between rural and urban older Oklahomans. Rural individuals were less likely get information from ageing agencies, churches, senior information lines, national organizations, the Internet, newsletters, fliers and television. In fact, the only sources of information that was higher for small town or isolated rural areas was getting information about the community from family, neighbours or friends. Similar to advice offered in 2003 by Scala [28] programs will be less effective in providing information to older Oklahomans in rural areas, but that persistence and using all of the resources combined (such as television and the Internet) were effective, but not as effective as in urban areas. Scala’s advice of needing assistance for finding services, recruiting local leadership, making connections and understanding the power of the word of mouth (family and friends) is still critical for these populations [28]. While we can and do still use all resources available we need to understand the uniqueness of the rural area and how people learn about services.

Strengths of this survey include the identification of senior interests and barriers to current programs for urban, sub-urban and rural adults in Oklahoma, which can be used guide for development and implementation of new senior programs into Oklahoma communities. Implementing such programs could potentially decrease health problems and increase quality of life among Oklahoma’s older adults. Barriers to programs identified by this survey can help determine methods to increase participation in newly implemented programs in specific rural or urban areas. We anticipate that additional analyses of the survey data will aid in appropriate methods of reaching Oklahoma seniors with advertisements that emphasize certain desired programs such as legal aid and tax preparations, in addition to health services, classes and activities. Finally this survey did include an adequate sample size for specific sub-analyses including rural and isolated areas.

Limitations of this study include the using voter registry as a population source and the somewhat low response rates, in particular among the sub-urban areas. Participants were selected from the Oklahoma Voter Registration file and the estimated voter registration differed by age group (87.4% for ages 65–74 and 66.5% for age 75 and older). Consequently, results of this survey may not be representative of the entire Oklahoma senior population, in particular those not eligible to vote and those less likely to register to vote despite eligibility. This latter group may be less socially engaged and at increased risk for poor health [50]. Differences in interests and barriers to program access likely exist between those who responded and those who did not.

## Conclusion

Results of this survey may be beneficial in program planning for Oklahoma seniors. It is important to address identified barriers to program access when planning future programs. Introducing additional community activities and programs may decrease high rates of physical inactivity and other poor health habits and improve overall health for Oklahoma seniors.

Findings from this state wide survey have been reviewed and were integral for OHAI in terms of program planning for older Oklahomans. For example, we identified the need “word of mouth” recruitment in isolated and rural areas. We also found that one of our most important efforts need to be in recruiting individuals who may benefit from social engagement including some of our trainings and classes but just do not want to go. To address this, we developed a specific training that targets depression but in such a way that older Oklahomans are not offended by the program guide; this training is referred to as, “Healthy Brain, Healthy Mind”.

Healthy Brain, Healthy Mind is a mental health education program developed by the OHAI in response to a need for mental health education in a state where discussing mental health is often stigmatic and not a regular topic of discussion for many Oklahomans. In order to circumvent this stigma, Healthy Brain, Healthy Mind does not address mental health issues directly. Instead, the curriculum of Healthy Brain, Healthy Mind refers to common mental health issues using terms like “managing the blues”, “managing stress”, or “moving forward”.

We have determined that both rural and urban populations are interested in health promotion services but that both urban core and rural areas have barriers, principally lack of resources. Identified barriers to program access will be addressed when planning future programs. We have worked with groups in each location to provide convenient and accessible locations for services, classes and activities. Furthermore, we anticipate that introducing additional specific community services, classes and activities will decrease poor health behaviors and improve overall health for Oklahoma seniors. As a next step, we plan to implement additional activities and services and evaluate the impact of these programs on health of seniors.

## Figures and Tables

**Table 1:** Survey responses by rural urban commuting areas for Oklahomans age 65 and older, 2013.

Rural Urban Communing Areas	Sample	Completed	Response Rate
Urban core^1^	1094	323	29.5%
Sub-urban areas^1^	1818	108	5.9%
Large rural town^1^	1148	332	28.9%
Small town and isolated rural^1^	2302	386	16.8%
*Unknown*^2^	*-*	*99*	*7.9%*
Total	6362	1248	19.6%

1 percent of known;

2 percent of total.

**Table 2:** Number, percent, and standard error of transportation methods and participation in current activities by rural urban commuting area, Oklahomans, 2013.

	Urban Core			Sub-Urban			Large Rural Town			Small Town and Isolated Rural			*P* Value
	N	%	SE	N	%	SE	N	%	SE	N	%	SE	
Transportation													
Own/drive a car	288	89.6	1.78	98	91.5	2.91	299	91.3	1.95	344	90.9	1.73	0.88
Relative not living with me drives	24	7.7	1.58	6	5.6	2.40	14	5.2	1.55	24	4.7	0.97	0.41
Church or social service agency	12	3.7	1.10	1	0.3	0.35	11	4.5	1.53	7	2.0	0.89	0.09
Transportation service	6	1.9	0.81	0			6	1.9	0.97	7	1.9	0.82	
Friends drive	24	7.9	1.60	6	4.8	2.10	23	7.5	1.80	25	7.6	1.69	0.75
I ride public transportation	8	2.2	0.81	0			7	2.5	1.11	3	0.6	0.38	
* Walk or ride a bicycle/tricycle*	***20***	***6.3***	***1.40***	***2***	***0.7***	***0.49***	***26***	***8.3***	***1.91***	***15***	***4.1***	***1.22***	***0.009***
Apartment management	0	-	-	0	-	-	1	0.2	0.15	0	-	-	
Spouse or other person living with me drives	42	12.8	1.93	17	14.8	3.55	63	19.2	2.59	62	15.7	2.20	0.25
Don’t go	4	1.4	0.71	1	1.0	1.04	6	2.4	1.12	5	1.4	0.76	0.79
													
Attended free health information event in the past year	***66***	***21.5***	***2.41***	***15***	***15.5***	***3.86***	***77***	***23.7***	***2.87***	***56***	***14.0***	***2.08***	***0.04***
													
Where spend most of time when away from home													
Senior Center	13	3.8	1.10	7	6.7	2.67	23	7.8	1.86	33	9.1	1.81	0.08
Church/Faith Center	133	40.3	2.84	43	36.9	4.92	158	49.1	3.33	175	44.4	3.02	0.12
With friends and relatives	193	60.2	2.84	60	55.0	5.19	193	58.0	3.31	220	57.9	3.02	0.80
Traveling	79	24.6	2.49	26	22.3	4.23	86	25.3	2.88	87	21.9	2.50	0.81
* I don’t leave my home*	***20***	***7.0***	***1.52***	***2***	***0.7***	***0.49***	***17***	***4.3***	***1.20***	***24***	***6.3***	***1.49***	***0.02***
Other	94	29.4	2.64	28	30.5	4.89	78	24.6	2.90	84	25.0	2.74	0.48

% and SE are weighted

**Table 3:** Number, percent, and standard error of Oklahomans’ interest in services, classes, and activities and perceived barriers to accessing programs by rural urban commuting area, 2013.

	Urban Core			Sub-Urban			Large Rural Town			Small Town and Isolated Rural			P Value
Services	N	%	SE	N	%	SE	N	%	SE	N	%	SE	
Legal assistance (wills, power of attorney, medical powers of attorney, etc.)	168	51.6	2.90	53	47.7	5.20	169	50.8	3.33	204	56.6	3.00	0.42
Assistance with tax preparation	104	33.5	2.75	25	20.1	4.06	107	33.6	3.16	115	31.4	2.90	0.06
Telephone reassurance (daily check-in calls)	29	9.5	1.72	4	3.5	1.80	28	8.5	1.85	30	7.4	1.58	0.22
* Congregate meals at a center*	***23***	***7.2***	***1.52***	***12***	***10.4***	***3012***	***35***	***11.8***	***2.22***	***58***	***14.4***	***2.10***	***0.05***
Home delivered meals	30	9.4	1.70	7	6.2	2.44	22	7.6	1.85	45	11.6	1.95	0.35
Health screenings	115	35.5	2.77	53	42.8	5.07	115	33.4	3.07	138	36.1	2.93	0.43
Prescription assistance	75	24.0	2.49	30	26.7	4.57	77	23.8	2.85	125	30.2	2.72	0.35
Respite for caregivers	30	10.0	7.77	8	7.2	2.63	19	5.8	1.47	26	6.7	1.52	0.26
Other	23	6.7	1.41	11	11.4	3.37	17	6.6	1.80	28	8.2	1.75	0.43
**Classes**													
Health and wellness	131	41.3	2.88	38	35.8	5.01	124	38.5	3.24	137	34.5	2.88	0.40
Cooking	56	18.2	2.26	23	23.1	4.45	64	19.8	2.67	68	16.0	2.10	0.44
Nutrition	94	29.6	2.67	34	32.1	4.87	79	24.4	2.85	86	23.5	2.62	0.18
Exercise	150	47.1	2.91	48	46.0	5.21	136	41.1	3.27	143	38.2	2.98	0.17
Mental Health	38	12.1	1.88	13	13.2	3.60	42	12.7	2.20	49	12.6	2.00	0.99
Chronic Disease	40	12.5	1.92	12	1.05	3.18	43	12.2	2.14	41	9.7	1.68	0.73
Caregiver	31	9.9	1.75	8	5.7	2.18	21	6.6	1.62	29	7.3	1.54	0.32
Using the computer and/or Internet	128	40.3	2.87	46	43.9	5.18	127	35.6	3.08	171	44.7	3.03	0.25
Arts and crafts/hobby	95	30.2	2.68	31	32.1	4.94	86	27.2	2.98	116	29.9	2.77	0.83
Storm preparedness (Tornado, Ice etc.)	37	12.4	1.95	9	8.4	2.83	43	11.0	1.92	62	14.1	1.95	0.45
Other (please specify)	22	6.8	1.45	4	3.6	1.85	9	3.3	1.32	17	4.9	1.33	0.30
**Activities**													
Indoor exercise activities	153	48.6	2.92	54	49.1	5.22	139	44.3	3.31	161	40.2	2.97	0.21
Outdoor exercise activities	82	26.3	2.56	38	35.5	5.01	74	23.9	2.84	86	22.8	2.58	0.09
Walking classes, supervised walking, walking trails	108	34.6	2.78	38	36.0	5.01	102	31.1	3.08	104	27.0	2.69	0.22
Card, board, and table games (bridge, poker, dominoes, scrabble, bingo etc.)	74	23.5	2.48	22	21.9	4.38	77	23.0	2.76	89	23.3	2.60	0.99
Day trips (such as to museums, parks)	116	35.9	2.80	42	37.4	2.80	138	43.3	3.30	157	40.8	2.99	0.35
Dances or dance lessons	50	15.9	2.13	19	20.1	4.29	44	14.0	2.36	53	13.9	2.14	0.48
Nature-related activities	70	21.7	2.39	29	25.6	4.49	78	26.0	2.95	72	19.0	2.41	0.30
Billiards, Shuffleboard, Ping Pong	26	8.3	1.62	11	10.8	3.22	20	5.8	1.54	28	8.0	1.70	0.49
Croquet, lawn polo, horseshoes Tennis, basketball, volleyball	30	9.2	1.67	12	10.8	3.15	27	9.2	1.97	24	706	1.77	0.81
**Other**	25	7.8	1.56	8	7.5	2.75	21	7.2	1.83	17	4.2	1.16	0.39

% and SE are weighted

**Table 4:** Barriers to accessing programs by rural urban commuting area status, Oklahoma, 2013.

	Urban Core			Sub-Urban			Large Rural Town			Small Town and Isolated Rural			P Value
	N	%	SE	N	%	SE	N	%	SE	N	%	SE	
*Transportation*	***28***	***10.9***	***2.00***	***5***	***5.2***	***2.47***	***12***	***4.0***	***1.39***	***16***	***4.1***	***1.22***	***0.004***
**Location**	43	15.8	2.28	24	23.7	4.58	41	15.5	2.63	83	22.7	2.65	0.09
*Lack of adequate facilities*	***22***	***7.8***	***1.68***	***15***	***13.9***	***3.70***	***40***	***15.9***	***2.70***	***60***	***16.4***	***2.28***	***0.01***
**Didn’t know about services**	67	23.2	2.61	27	28.9	5.01	67	23.6	2.98	54	16.6	2.47	0.09
**Don’t know how to access/enroll in services**	24	8.5	1.73	8	7.9	2.95	18	6.0	1.64	24	7.8	1.85	0.81
**Just don’t want to go**	71	26.0	2.75	19	19.9	4.39	88	33.9	3.47	106	31.6	3.00	0.05
**Other**	64	22.7	2.63	18	20.2	4.50	58	21.3	3.00	58	18.0	2.53	0.66

% and SE are weighted

**Table 5:** Resources for finding help for older adults and resources in the respondents’ community by rural urban commuting area status, Oklahoma, 2013.

	Urban Core			Sub-Urban			Large Rural Town			Small Town and Isolated Rural			P Value
	N	%	SE	N	%	SE	N	%	SE	N	%	SE	
**Help for older adults**													
*** Aging agencies, senior centers, or retirement communities***	***68***	***21.5***	***2.40***	***7***	***6.3***	***2.48***	***59***	***14.6***	***2.15***	***57***	***14.4***	***2.11***	***0.0008***
*** Church***	***108***	***34.4***	***2.77***	***32***	***25.8***	***4.37***	***117***	***33.8***	***3.09***	***101***	***24.9***	***2.59***	***0.04***
Community organizations	39	12.6	1.96	12	10.8	3.15	52	16.1	2.46	42	10.9	1.96	0.39
Doctor’s office, VA clinic, or registered nurse	108	33.5	2.75	39	32.7	4.80	107	34.8	3.21	145	36.9	2.92	0.83
*** Senior Infoline***	***18***	***6.0***	***1.42***	***6***	***7.1***	***2.81***	***4***	***0.6***	***0.30***	***7***	***2.1***	***0.96***	***0.0009***
Family members, neighbors, or friends	147	47.0	2.91	54	48.2	5.22	151	46.9	3.31	195	52.2	3.03	0.63
Government agency	27	8.0	1.58	4	4.7	2.33	15	4.0	1.24	24	6.5	1.56	0.28
Internet	112	35.5	2.79	29	28.0	4.69	83	25.5	2.89	100	28.6	2.83	0.07
*** National organizations (For Example AARP)***	***89***	***28.9***	***2.65***	***23***	***20.6***	***4.14***	***86***	***26.4***	***2.92***	***82***	***18.8***	***2.21***	***0.03***
Newspaper, magazines	173	54.7	2.88	63	56.0	5.21	171	51.7	3.32	198	51.4	3.03	0.77
Other (please specify)	24	7.2	1.49	9	8.3	2.89	15	6.0	1.75	29	7.3	1.54	0.90
													
**In your community**													
Church/Faith-based organization	139	43.3	2.88	49	38.5	4.92	168	50.7	3.32	198	49.3	3.03	0.10
Community center or other community group/organization	32	9.2	1.67	10	9.5	3.09	42	12.3	2.15	48	11.6	1.91	0.65
*** Family, neighbors, or friends***	***188***	***58.6***	***2.86***	***73***	***68.2***	***4.83***	***240***	***72.1***	***3.03***	***288***	***74.9***	***2.60***	***0.0002***
*** Internet***	***102***	***33.0***	***2.75***	***27***	***26.4***	***4.60***	***87***	***27.0***	***2.96***	***73***	***19.4***	***2.42***	***0.005***
*** Newsletters, flyers, or bulletins***	***140***	***45.7***	***2.90***	***41***	***40.0***	***5.10***	***137***	***42.9***	***3.29***	***125***	***33.9***	***2.90***	***0.04***
Newspaper	224	69.3	2.68	73	65.2	4.96	247	74.8	2.90	283	71.0	2.83	0.34
Radio	84	26.2	2.56	24	22.9	4.37	86	27.3	3.01	80	18.6	2.24	0.13
*** Television***	***235***	***72.0***	***2.63***	***66***	***56.1***	***5.15***	***198***	***62.2***	***3.20***	***195***	***51.5***	***3.03***	***<0.0001***
Other	13	4.2	1.17	4	3.6	1.82	11	3.2	1.17	14	3.6	1.15	0.95

% and SE are weighted

**Table 6:** Summary of Findings Oklahoma, 2013.

Demographics	There was no difference among the rural and urban groups by age, gender. Those living in small town and isolated rural areas were more likely than those living in any other area to live in poverty.
Transportation	Older adults in all cores reported that they own/drive a car and spend most of their time away from home with family.
Services	Most frequently reported services included legal assistance (high frequency across all cores) with slight differences in priority between second and third most reported for assistance with tax preparation and health screenings.
Classes	Need for exercise classes were the most frequently reported classes for urban, suburban, and large rural towns, but lower priority for small town/ isolated rural areas. Classes focused on health and wellness and using the computer/internet were reported second or third most frequently.
Activities	Indoor exercise activities were reported most frequently by urban, suburban, and large rural towns, whereas day trips and walking activities were reported second and third most frequently (though reported most frequently for small/isolated rural areas).
Health Info	Information from newspaper/magazines was reported most frequently for urban, suburban, and large rural towns, whereas information from family/ friends, healthcare providers, or internet was reported less frequently. Information from family/friends was reported most frequently by small/isolated rural areas.
Community Info	The trends were less clear for sources of community information with the top three resources varying for each area, including information from family/friends, newspaper, or television.
